# Managing HIV/hepatitis C co-infection in the era of direct acting antivirals

**DOI:** 10.1186/1741-7015-11-234

**Published:** 2013-11-01

**Authors:** Jürgen K Rockstroh, Sanjay Bhagani

**Affiliations:** 1 Department of Medicine I, University Hospital Bonn, Sigmund-Freud-Str. 25, 53105 Bonn, Germany; 2 Department of Infectious Diseases/HIV Medicine, Royal Free London, NHS Foundation Trust, London, UK; 3 Research Department of Infection, UCL, London, UK

**Keywords:** HIV, Hepatitis C, Direct acting antivirals, Interferon, Ribavirin

## Abstract

Morbidity and mortality from co-morbid hepatitis C (HCV) infection in HIV co-infected patients are increasing; hence, the management of hepatitis co-infection in HIV is now one of the most important clinical challenges. Therefore, the development of direct acting antivirals (DAAs) for treatment of HCV has been eagerly awaited to hopefully improve HCV treatment outcome in co-infected individuals. Indeed, the availability of the first HCV protease inhibitors (PI) boceprevir and telaprevir for HCV genotype 1 patients has changed the gold standard of treating hepatitis C allowing for substantially improved HCV cure rates under triple HCV-PI/pegylated interferon/ribavirin therapy. Moreover, numerous other new DAAs are currently being studied in co-infected patient populations, also exploring shorter treatment durations and interferon-free treatment approaches promising much easier and better tolerated treatment regimens in the near future. Nevertheless, numerous challenges remain, including choice of patients to treat, potential for drug-drug interactions and overlapping toxicities between HIV and HCV therapy. The dramatically improved rates of HCV cure under new triple therapy, however, warrant evaluation of these new treatment options for all co-infected patients.

## Introduction

The introduction of highly active antiretroviral therapy (HAART) in 1996 and the resultant sustained control of HIV replication and consequent immune reconstitution, has decreased HIV associated morbidity and mortality dramatically. Consequently, liver disease, mostly as a result of chronic viral hepatitis co-infection, has emerged as one of the most important causes of non-AIDS associated morbidity and mortality in HIV infected patients [1]. HIV and hepatitis C viruses share similar rules of transmission leading to a high rate of hepatitis C co-infection among patients with HIV. Overall it is estimated that around 25% of all European HIV patients have concomitant hepatitis C virus (HCV) co-infection [2,3]).

In the natural course of hepatitis C in HIV, in the absence of antiretroviral therapy, a faster progression of hepatic fibrosis has been observed. This is particularly true in patients who develop CD4 counts below 200 cells/mm^3^ or develop AIDS [4]. This enhanced fibrosis progression may be a result of the direct fibrogenetic effect of HIV in the liver and a likely consequence of HIV induced impairment of the innate and adaptive immune system leading to more inflammation, apoptosis and fibrosis [5]). Importantly, successful anti-HIV therapy can slow down the accelerated cause of fibrosis progression in HIV/HCV co-infected patients, emphasizing the importance of maintaining high CD4 counts in these patients [6]). However, despite attenuation of faster fibrosis progression with successful HAART, some patients continue to develop hepatocellular carcinoma and advanced liver disease. Therefore, the treatment of HCV is of the utmost clinical importance. Although the introduction of pegylated interferon (pegIFN) and ribavirin (RBV) combination therapy simplified HCV treatment and was associated with improved response rates, the outcome of treatment remained unsatisfying particular for genotype 1 and 4 patients where cure rates defined as sustained virological response (negative HCV PCR 24 weeks after stopping therapy) (SVR) was not higher than 22 to 35% [7-9]. The development of direct acting antivirals (DAAs) against HCV promising higher cure rates in this challenging patient population have led to substantial hope that better treatment options have finally arrived. In 2011, the Food and Drug Administration (FDA) approved the use of the first two DAAs, boceprevir and telaprevir, for the treatment of HCV genotype 1 infection in mono-infected patients. Subsequently, the first pilot trials with the use of these compounds in HCV treatment naïve as well as HCV treatment experienced HIV/HCV co-infected patients have been presented. To date, the results of the studies suggest excellent treatment response rates with SVRs in the range of 60 to 80% and an increase in SVRs by 29 to 35% when pegIFN and RBV were used as comparators [10-13]. Despite this, treatment of HCV in HIV remains complex with multiple challenges, including high pill burden, higher rates of adverse events (AEs) and difficult drug-drug-interactions commonly seen between HIV drugs and HCV protease inhibitors that need to be addressed. From a clinician’s perspective this calls for practical clinical algorithms which could help in the day-to-day management of these patients. This review aims at summarizing the currently available data on treatment of chronic HCV with DAA-based therapy and to provide guidance and whom to treat now and where to wait for improved options in the near future. Management of acute HCV is outside the scope of this article.

### Pegylated interferon and ribavirn therapy in HIV/HCV co-infection

**Figure 1:**
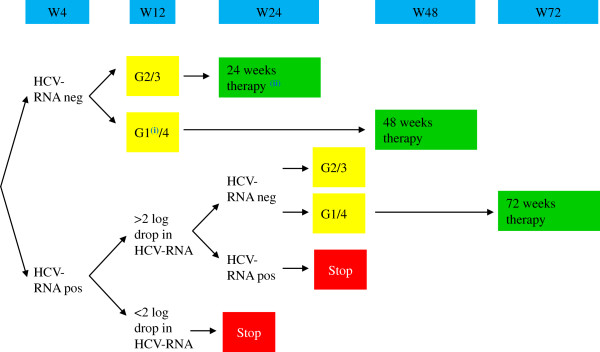
**Optimal duration of dual HCV therapy in HCV/HIV co-infected patients not eligible for triple therapy.** (adapted with permission from http://www.europeanaidsclinicalsociety.org/). Legend: i) Where no access to DAA is available or high chances of cure even with dual therapy (favorable IL28B genotype, low HCV viral load and no advanced fibrosis). ii) In patients with baseline low viral load (<600,000 IU/mL) and minimal liver fibrosis. DAA, direct acting antivirals; HCV, hepatitis C virus.

Until recently, dual therapy with pegIFN and RBV has been the gold standard for HCV therapy in HIV co-infected individuals. Overall SVR rates have been between 25 to 50%, with cure rates approaching 70 to 80% in HCV genotype 2 or 3 infection and considerably lower at between 18 to 38% in HCV genotype 1 and 4 patients [14]. Treatment uptake, however, was very low, mostly due to the fear of high adverse event rates (in particular, central nervous system (CNS) toxicity) associated with pegIFN/RBV combination therapy, a high rate of comorbid medical and psychiatric conditions representing a contraindication for dual HCV therapy coupled with high cost of therapy and a high proportion of genotype 1 infections with low SVR rates. Indeed, data from the EuroSIDA cohort suggest that not more than 25% of all European HIV/HCV co-infected patients have ever received anti-HCV therapy [15]). However, it needs to be emphasized that cohort studies evaluating the clinical outcome of HCV therapy have demonstrated that achieving SVR is associated with a significant decline in risk of liver-specific and all-cause mortality in patients with HCV mono-infection as well as in HIV/HCV co-infection. In the Veterans Affairs HCV mono-infection population, SVR was associated with a marked reduction in all-cause mortality [16]). Similarly, in the Spanish GESIDA HCV/HIV cohort, SVR was accompanied by a significant decrease in liver-related complications and mortality as well as non-liver-related mortality [17,18]. Interestingly, in more recent analyses, HIV/HCV co-infected patients with end-of-treatment response but relapse after stopping dual HCV therapy developed less liver-related mortality, decompensations and liver stiffness progression than treatment non-responders, suggesting a benefit from successful HCV viremia suppression to the end of a finite treatment course, even if a cure was not achieved [19]. Therefore, for HIV patients with genotype 2,3 and 4 where no licensed DAAs are currently available, dual HCV therapy with pegIFN and RBV remains the gold standard. The current treatment algorithm for these HCV genotypes is summarized in Figure 1.

### Direct Acting Antivirals (DAA) in HIV/HCV co-infection

Resolution of the three-dimensional structures of several HCV proteins, together with the development of replicative cell culture systems, has led to the identification of a number of potential targets for DAAs [20]. Numerous families of drugs that potently inhibit the HCV life cycle *in vitro* have been identified, and many of these molecules have reached early to late clinical development. Two NS3-4A protease inhibitors, telaprevir and boceprevir, were the first DAAs to become approved in Europe and the United States in 2011 in combination with pegIFN-α and RBV for the treatment of HCV genotype 1 infection. Numerous other DAAs are at the clinical developmental stage in combination with pegIFN and RBV or with other DAAs in IFN-free regimens, with or without ribavirin. They include first-generation, second-wave and second-generation NS3-4A protease inhibitors, NS5B polymerase inhibitors, inhibitors of non-structural protein 5A and host-targeted agents, such as cyclophilin A inhibitors and microRNA-122 antagonists.

### Currently licensed DAAs for HCV therapy in treatment-naïve HIV/HCV patients

Recently, two pilot studies, one with boceprevir- and one with telaprevir-based HCV therapy, have been published with full SVR24 results in treatment-naïve HIV/HCV co-infected patients and are briefly summarized below [10,11].

A phase IIa, double-blind study in 98 HCV/HIV co-infected patients investigated the safety and efficacy of boceprevir in combination with PegIFN-α-2b and weight-based dose ribavirin [10]. Patients were randomly allocated (1:2) according to a computer generated sequence, stratified by Metavir score and baseline HCV RNA level, to receive pegIFN-alpha-2b 1.5 μg/kg per week with weight-based RBV (600 to 1,400 mg per day) for four weeks, followed by pegIFN/RBV plus either placebo (control group) or 800 mg boceprevir three times per day (boceprevir group) for 44 weeks. All patients were HCV-treatment naïve and had genotype 1 HCV infection. The majority of patients included in the study were male (69%), Caucasian (82%), non-cirrhotic (95%) and had a HCV viral-load >800,000 IU/ml (88%). In addition, they were mostly on HAART, with an undetectable HIV viral load and CD4 cell count >500 cells/mm^3^. The antiretroviral therapeutic combinations used in the study were predominately based on boosted protease inhibitor (PI) (>90%). Non-nucleoside reverse-transcriptase inhibitors, zidovudine and didanosine were not permitted. A total of 40 (63%) of 64 patients in the boceprevir group had an SVR at follow-up Week 24, compared with 10 (29%) of 34 control patients (difference 33.1%, 95% CI 13.7 to 52.5; *P* = 0.0008). Adverse events were more common in patients who received boceprevir than in control patients: 26 (41%) versus 9 (26%) had anemia, 23 (36%) versus 7 (21%) pyrexia, 22 (34%) versus 6 (18%) had decreased appetite, 18 (28%) versus 5 (15%) dysgeusia, 18 (28%) versus 5 (15%) vomiting, and 12 (19%) versus 2 (6%) neutropenia. Three patients who received boceprevir plus pegIFN/RBV and four controls had HIV virological breakthrough.

A phase IIa, randomized, double-blind, placebo-controlled study in 62 HCV/HIV co-infected patients investigated the safety and efficacy of telaprevir in combination with PegIFN-α-2a and 800 mg RBV (US) and weight-based dose RBV in France and Germany [11]). A total of 62 patients with HCV genotype 1 infection and HIV-1 infection who were HCV treatment-naive were enrolled in the study. Patients were required to be receiving no antiretrovirals (part A) or one of two specified antiretroviral regimens (part B), including either efavirenz or ritonavir boosted atazanavir. Patients in part A were randomly assigned in a 1:1 ratio and patients in part B were randomly assigned in a 2:1 ratio to receive telaprevir or placebo, both in combination with PEG-IFN-α-2a and ribavirin for 12 weeks, plus 36 weeks of PEG-IFN-α-2a and ribavirin. Analyses of all patients from the different treatment arms showed that SVR occurred in 74% (28 in 38) of patients receiving telaprevir plus PEG-IFN-α2a/RBV and 45% (10 in 22) of patients receiving placebo plus PEG-IFN-α-2a/RBV. Rapid HCV suppression was seen with telaprevir plus PEG-IFN-α-2a + ribavirin (68% (26 in 38 patients) vs. 0% (0 in 22 patients) undetectable HCV RNA levels by Week 4). Two patients had on-treatment HCV breakthrough with telaprevir-resistant variants. Patients treated with antiretroviral drugs had no HIV breakthroughs; antiretroviral exposure was not substantially modified by telaprevir. Pruritus, headache, nausea, rash and dizziness were higher with telaprevir plus pegIFN-α2a/RBV during the first 12 weeks. During this period, serious adverse events occurred in 5% (2 in 38) of those receiving telaprevir plus pegIFN-α-2a in combination with RBV and 0% (0 in 22) of those receiving placebo plus pegIFN-α-2a and RBV; the same number in both groups discontinued treatment due to adverse events.

In summary, both pilot trials in naïve HIV/HCV co-infected patients have demonstrated statistically superior treatment outcome responses in patients receiving DAA-based HCV therapy over dual therapy with pegIFN/RBV alone. Therefore, within the European AIDS Clinical Society (EACS) hepatitis co-infection guidelines (please also see http://www.europeanaidsclinicalsociety.org/), HCV treatment in HCV genotype 1 patients is recommended with triple therapy, including either of the HCV protease inhibitors boceprevir or telaprevir in combination with pegIFN and RBV where available.

### First licensed DAAs in HCV treatment experienced HIV/HCV patients

At the Conference on Retroviruses and Opportunistic Infections (CROI) 2013 meeting in Atlanta, first results were presented from two Agence Nationale de Recherche sur la SIDA (ANRS) studies which looked at the efficacy and safety of triple HCV therapy (either with telaprevir or boceprevir) in previous non-responders to dual pegIFN/RBV therapy [12,13]. Patients with previous null-response and cirrhosis were excluded because of the overall low probability of treatment response. Both studies were presented as pre-planned interim analyses at Week 16 of therapy. In the telaprevir ANRS HC26 study at the interim analyses, 69 patients who received at least one dosage were included [12]. Background HIV therapy contained atazanavir (ATV), ritonavir-boosted atazanavir (ATV/r), efavirenz (EFV), raltegravir (RAL), tenofovir (TDF), emtricitabine (FTC) or lamivudine (3TC). Patients started with a four-week lead-in of pegIFN/RBV. This approach, which is outside the labeling of telaprevir, was chosen so the investigators could compare the results to the boceprevir study, which had a lead-in period of four weeks and because data from telaprevir studies in HCV mono-infection in treatment experienced patients had potentially promised some benefit for this approach. After Week 4, telaprevir was added for12 weeks. Patients who achieved a complete rapid virological response at Week 8 (RVR8) defined as HCV-RNA <15 IU/mL received 32 weeks of pegIFN/RBV after stopping telaprevir at Week 16 following12 weeks of triple therapy (full treatment: 48 weeks). Whereas patients who only obtained a partial RVR8 (15 IU/mL < HCV-RNA <1,000 IU/mL) received an additional 56 weeks of pegIFN/RBV (full treatment: 72 weeks). Telaprevir was administered as 750 mg q8h (1,125 mg q8h with EFV) and pegIFN α-2a as 180 μg sc/week. Ribavirin was dosed as 1,000 mg/day (≤75 kg) and 1,200 mg/day (>75 kg). Futility rules for telaprevir were HCV-RNA >1,000 IU/mL at Week 8 or Week 12 or virological breakthrough at any time. For study inclusion patients needed to have CD4 ≥200 cells/mm^3^ and ≥15%, as well as plasma HIV-RNA levels <50 copies/mL. At baseline, the median CD4-count was 630 cells/mm^3^ (range 459 to 736) and HIV-RNA was below 50 c/ml in 99% of patients. Eleven (18%) patients had Metavir F3 fibrosis and 16 (23%) had F4 fibrosis at inclusion into the study. A total of 39% of patients were previous relapsers to dual therapy, 9% had previous viral breakthrough, 22% were partial responders and 30% null-responders. With 88% achieving an undetectable HCV-RNA at Week 16 in this patient population comprising of 30% previous null-responders and 40% with F3/F4 fibrosis, this study has achieved surprising high early efficacy results. Most interestingly, efficacy remained high independent of a previous response to dual HCV therapy (early virological response at Week 16 (EVR_16_) was 86% in relapsers versus 86% in previous null-responders) or baseline fibrosis stage (EVR16 was 92% in F1 and 94% in F4 patients). Concomitant antiretroviral therapy (ART) also had no impact on early virological response. Adverse events were frequent (99% of patients developed an AE) but were mostly related to pegIFN side effects. A total of 4% (n = 3) of patients discontinued study drugs because of psychiatric adverse events and cutaneous adverse events, respectively. Noteworthy, in contrast to the pilot trial in HCV treatment-naïve patients where no telaprevir discontinuation because of rash was recorded, a few discontinuations because of rash did occur in this study. Grade 3 to 4 anemia, erythropoietin (EPO) use, transfusion or RBV dose reduction was recorded in 61% of patients.

The second ANRSstudy looked at the efficacy and safety of a boceprevir containing triple therapy in 64 previous IFN/RBV non-responders [13]. Following a lead-in with dual therapy, boceprevir was added at Week 4. Boceprevir was discontinued in all patients with a HCV viral load above 1,000 IU/ml at Week 8 and/or at Week 12. All HCV drugs were discontinued if HCV viral load was >1,000 IU/ml at Week 16 or still detectable at Week 28 or in case of virological breakthrough. Patients who achieved a complete rapid virological response at Week 8 (RVR8) defined as HCV-RNA <15 IU/mL received another 40 weeks of triple therapy. Patients who at Week 8 had an HCV viral load >15 IU/mL but <1,000 IU/mL triple therapy continued for four more weeks. If their HCV viral load at Week 12 was again >15 IU/mL but <1,000 IU/mL, they received an additional 36 weeks of triple therapy followed by a further 12 weeks of pegIFN/RBV. Patients recruited for the trial had to be on stable ART for at least three months, with at least three molecules among ATV (ritonavir boosted or not), RAL, TDF, ABC, FTC or 3TC. Again, only previous non-responders to dual therapy with HCV genotype 1 infection were included. Overall, 17% of patients had Metavir F4 fibrosis at baseline and 33% were previous null-responders. Overall, 63% achieved undetectability at Week 16, which was slightly higher in the RAL-treated patients than in patients on other ART regimens. Response rates at Week 16 were best for previous relapsers (with 90% <15 IU/ml) versus patients with previous breakthrough (60% <15 IU/ml), partial responders (60% <15 IU/ml) and null-responders (38% <15 IU/ml), respectively. Response rates according to fibrosis at baseline in contrast were comparable between the different fibrosis stages (62% <15 IU/ml at Week 16 with F1, 67% for F2, 50% for F3 and 73% for F4). Anemia occurred in 42% of patients but only 5% developed grade 3 to 4 anemia. A total of 42% of the patients received concomitant EPO.

Both of these studies show very impressive early treatment response rates independent of baseline fibrosis stage (please note that patients with prior null-response and F4 fibrosis, however, were excluded from the trial). Whether the good early response rates can be maintained and translate into good SVR rates remains to be seen, as at this time these studies are still ongoing. It is noteworthy, however, that triple therapy studies in HCV treatment-experienced patients (see the ANRS CO20-CUPIC trial) also showed good early treatment response rates, which, however, did not translate to high SVR rates after the end of therapy [21]. Moreover, similar to CUPIC, significant hematological toxicity was observed in these studies despite proactive management of anemia. However, significant drop-out rates as a result of SAEs were not seen by Week 16. Moreover, in the CUPIC trial, there was a considerable incidence of severe complications and death, especially in patients with low platelet counts and low albumin. This has not as yet been reported in the early results from the ANRS co-infection trials but needs to be borne in mind with respect to treating patients with advanced fibrosis/cirrhosis with currently available triple therapy. These patients should be managed withincenters with experience in looking after co-infected patients with advanced liver disease.

### Trial results with new DAAs in HIV/HCV co-infected individuals

At present, several other HCV trials are currently taking place in the HIV/HCV co-infected patient population and early treatment response rates from these studies have already been presented at conferences. Among the DAA anti-HCV drugs, the three most likely to be licensed in 2014 is the once-daily nucleotide analogue HCV polymerase inhibitor sofosbuvir, as well as the second-wave, once-daily HCV protease inhibitors simeprevir and faldaprevir. For both HCV protease inhibitors, data from co-infection trials are available and summarized below [22,23].

STARTVerso4 is an open-label, sponsor-blinded study in HCV/HIV co-infected patients who were HCV treatment-naïve (TN) or relapsed after previous HCV therapy to assess the efficacy and safety of faldaprevir q.d. plus pegIFN/RBV, and to evaluate 24-week treatment duration in HIV/HCV co-infected patients [22]. Patients in arm A were treated with faldaprevir at 120 mg/pegIFN/RBV for 24 weeks; Arm B: faldaprevir at 240 mg/PegIFN/RBV for 12 weeks then re-randomization at Week 12 to a further 12 weeksof faldaprevir/pegIFN/RBV or pegIFN/RBV alone. For response-guided therapy, early treatment success (ETS) was defined as a HCV RNA below the limit of quantitation at Week 4 and below the limit of detection at Week 8. At Week 24, ETS were re-randomized to stop treatment at Week 24 or continue pegIFN/RBV through Week 48. Patients without ETS received pegIFN/RBV through Week 48. Patients on HIV protease-based ART or efavirenz were allocated to faldaprevir 120 mg or 240 mg q.d., respectively; those receiving other allowed ART (raltegravir or maraviroc) or no ART were randomized to either dose. The primary endpoint of the study is SVR12. Overall, 304 (239 treatment naïve and 69 relapsers) patients were included, making this the largest co-infection DAA study so far. Importantly, 17% of the patients included had F4 fibrosis, 78% GT1a and baseline HCV RNA was ≥800,000 IU/mL in 80% of patients.

Early virological treatment response up to Week 12 was excellent in naïve as well as in previous relapsers. ETS was observed in 80% of patients with half of these patients being able to stop treatment at Week 24. This study will provide very interesting data on potentially shorter treatment durations in HIV/HCV co-infected subjects for the first time in the near future. The adverse event profile was comparable to adverse events observed with faldaprevir and pegIFN/RBV in HCV mono-infected patients [23]. A total of 18% of patients developed anemia and 18% a rash. Two rashes were documented as SAEs.

The other second wave HCV protease inhibitor study evaluated efficacy and safety of simeprevir based triple therapy in HIV/HCV co-infection in study C212. C212 is a phase III, open-label, single-arm, international trial assessing simeprevir (150 mg q.d.) plus pegIFN/RBV in treatment-naive and -experienced patients (N = 106) co-infected with genotype-1 HCV and HIV-1 [24]. Patients who were treatment-naïve and non-cirrhotic (n = 50) or prior relapsers (n = 14) received a response-guided treatment (RGT) regimen of simeprevir (150 mg once daily) for 12 weeks plus pegIFN/RBV for 24 or 48 weeks. Prior partial (n = 10) or null (n = 28) responders and patients with cirrhosis (three treatment-naïve patients and one relapser) received treatment for 48 weeks. The primary endpoint of the study was again SVR12, as well as safety and tolerability. Combination HCV therapy with simeprevir 150 mg q.d. + pegIFN/RBV led to high virologic response rates in co-infected patients, regardless of prior response (SVR12 77% in treatment-naïve and prior relapsers). Relapse occurred only in patients infected with HCV genotype-1a: 5/31 overall population; 3/22 treatment-naive; 2/9 prior relapsers. A total of 64% of all prior null responders had not experienced failure at the time of the interim analysis. Of the 88% of patients who met RVR, 75% achieved SVR 12; 30% of patients developed grade 3 or 4 events. A rash was reported for 17% of the patients. Hyperbilirubinemia (a known side effect of simeprevir) was noted in 5% of study subjects. However, only 4% of patients discontinued simeprevir because of adverse events. Overall, simeprevir was well tolerated, with a safety profile similar to that in HCV mono-infected patients.

In summary, these trials suggest that simplified DAA-based HCV therapy will become available very shortly, promising for a smaller tablet burden, with at least equal efficacy compared to the current triple therapy regimens but with better tolerability. Further co-infection studies include studies with daclatasvir, a NS5A inhibitor, as well as the first interferon-free studies with sofosbuvir. No interim results from these studies, however, have been publically presented to date.

### Who to treat now, where to wait?

**Figure 2:**
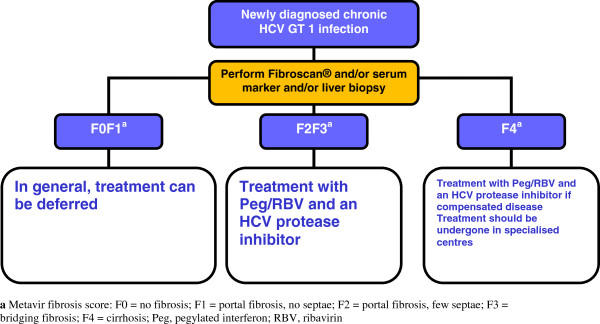
**Management of newly diagnosed HIV/HCV co-infected genotype 1 patients (adapted with permission from **[25]**).** Legend: Metavir fibrosis score: F0 = no fibrosis; F1 = portal fibrosis, no septae; F2 = portal fibrosis, few septae; F3 = bridging fibrosis; F4 = cirrhosis; Peg, pegylated interferon; RBV, ribavirin.

**Figure 3:**
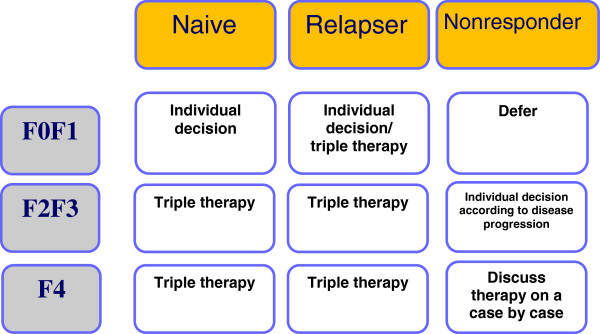
**Management of HIV-HCV co-infected genotype-1 patients by fibrosis stage/prior treatment (adapted with permission from **[25]**).**

With the rapid advances in HCV therapy making simplified and better tolerated HCV therapy with higher cure rates a realistic vision within the next two years, current treatment algorithms need to balance the need for preventing liver disease progression and occurrence of hepatocellular carcinoma against the side effects of current therapies and improved treatment options in the near future. Therefore, the first essential step in HCV treatment decision-making is fibrosis stage assessment, as the current fibrosis stage of a patient reliably predicts subsequent clinical risk of developing relevant liver disease. In patients with low fibrosis stages, treatment can be safely deferred as risk of liver disease progression, particularly in the setting of controlled HIV replication, is low and, therefore, patients could wait for newer treatment options. Patients with more advanced liver fibrosis stages, however, are at risk of developing more severe liver disease associated complications and can be potentially cured now with the currently available DAA-based treatment regimens. Figure 2 shows the management algorithm of persons with newly diagnosed HCV infection, which is adapted from the EACS guidelines [25]). Besides the fibrosis stage, previous response to interferon- and ribavirin-based dual HCV therapy is also important information in the decision making process. All studies to date have shown that relapsers, in particular, are most likely to benefit from DAA-based HCV re-treatment and, therefore, are preferred candidates, whereas patients with previous null-response (defined as less than 2 log drop in HCV-RNA levels under pegIFNIFN/RBV) therapy are much less likely to respond. Waiting for more potent DAA combinations is probably the best strategy for this group if fibrosis stage allows this (see Figure 3). It is important to bear in mind that if this is the case, careful monitoring of fibrosis progression would be essential.

While a number of new DAA-based therapies and IFN-free therapies, including combinations within fixed-drug combination tablets are in phase 2 and phase 3 clinical trials for mono-infected patients, there are an increasing number of studies underway or in the planning stage for co-infected patients. These include the IFN-free combination of sofosbuvir and ribavirin for G1, 2, 3 and 4 patients, a combination of Peg-IFN-lamda (a potentially better tolerated IFN) with ribavirin and the NS5a inhibitor daclatasvir also for G1, 2, 3 and 4 infections and ABT-450/r, a ritonavir-boosted protease inhibitor together with NS5a inhibitor ABT-267, non-nucleoside polymerase inhibitor ABT-333 combined with ribavirin for G1 patients. There is no doubt that better-tolerated, more efficacious and potentially IFN-free regimens are on the horizon for co-infected patients.

### Practical recommendations: CD4-count level and drug-drug interactions

Cure rates of HCV therapy in HIV co-infection have been demonstrated to increase with higher CD4 relative percentage above 25%, and undetectable HIV-RNA has been suggested to be independently associated with improved SVR rates ([26]. Therefore, in patients with a CD4-count <500 cells/mm^3^ early ART initiation is recommended to optimize HCV treatment outcome. Only in patients with a CD4-count above 500/μl without HIV therapy should HCV treatment in the absence of HIV therapy be considered. This strategy offers the advantage of decreased pill burden, lack of overlapping toxicities between HIV and HCV drugs and no drug-drug interactions.

As HCV protease inhibitors, as well as HIV protease inhibitors and NNRTIs, are all metabolized by the cytochrome p450 pathway, multiple complex drug-drug interactions exist between HCV and HIV drugs. Table 1 summarizes the corresponding drug interactions. Telaprevir can currently only be safely combined with boosted atazanavir, raltegravir, maraviroc, rilpivirine, etravirine or efavirenz (with EFV, telaprevir doses need to be increased to 1,125 mg every eight hours) in combination with tenofovir or abacavir and FTC or 3TC (please also check http://www.hep-druginteractions.com). Due to drug-drug interactions, boceprevir can only be currently safely combined with raltegravir, rilpivirine or etravirine in combination with tenofovir or abacavir and FTC or 3TC. The European Medicines Agency (EMEA) has also suggested considering boceprevir in combination with boosted atazanavir in patients with no previous HIV treatment failure and no drug resistance who have suppressed HIV-RNA when starting HCV therapy as boceprevir exposure is not impacted by concomitant boosted atazanavir, whereas atazanavir area under the curve (AUC) decreased significantly but trough levels remained above the recommended IC90 in all patients. It is not possible to co-administer simeprevir with HIV protease inhibitors. Faldaprevir can be combined with ritonavir boosted darunavir but the dose needs to be reduced to 120 mg once a day. So far, first results from interaction studies with sofosbuvir, which is not metabolized by the cytochrome p450 pathway, indicate that this compound can be combined with darunavir/r, atazanavir/r or efavirenz [27], thereby apparently offering more HIV treatment choices along with HCV therapy.

**Table 1:** Summary of key drug-drug interactions between HIV drugs and the licensed DAAs and dosing recommendations

	**Telaprevir**	**Boceprevir**
**ATV/r**	Monitoring for hyperbilirubinemia recommended	Consider on a case by case basis if deemed necessary
**DRV/r/, FPV/r LPV/r**	Not recommended	Not recommended
**EFV**	Increase TVR to 1,250 mg q8h	Not recommended
**ETR**	No dose adjustment needed	No dose adjustment needed
**RPV**	No dose adjustment needed	No dose adjustment needed
**RAL**	No dose adjustment needed	No dose adjustment needed
**TDF**	Increased monitoring is warranted	No dose adjustment needed

Taking into consideration all the complex issues surrounding anti-HCV treatment in this group of patients, particularly drug-drug interactions and side-effects, it is important that these patients are managed in centers with experience in managing HCV/HIV co-infected patients. Many physicians may want to start HCV/HIV co-infected patients on cART therapies that will have the least potential for drug-drug interactions, although it would be possible to switch cART during the period of DAA-based therapy. It is important to ensure that all co-medications, and not just cART components, that patients are on are documented and scrutinized for potential drug-drug interactions with the new DAAs. It is also imperative that as many patients as possible are included in the forthcoming clinical trials.

## Conclusion

HCV therapy has also dramatically changed with the advent of the first DAAs in the HIV/HCV co-infected population with the promise of much higher cure rates and potentially shortened treatment durations. Nevertheless, complex drug interactions as well as significant additional toxicities and challenging overall management issues have limited the uptake of these new treatment strategies to date. With easier to take, better tolerated and optimally interferon-free HCV treatment approaches on the horizon in the very near future, HCV treatment initiation can be delayed in all patients with low fibrosis stages. For patients with more advanced fibrosis, however, the new treatment modalities should at least be discussed in order not to miss out on further liver disease progression and HCC development.

## Competing interests

In the last five years, Jürgen Rockstroh has received honoraria for speaking at educational events or for consulting for Abbott, Abbvie, Astella, Bionor, BMS, Boehringer Ingelheim, Gilead, Janssen, Merck, Novartis, Pfizer, Vertex and ViiV.

In the last five years, Sanjay Bhagani has received honoraria for speaking at educational meetings, attending advisory meetings or travel support for attending conferences from Abbvie, BMS, Boehringer Ingelheim, Gilead, Janssen, MSD and Roche.

## Authors’ contributions

JKR and SB have both written this review and both authors read and approved the final manuscript.

## Authors’ information

JKR is a Professor of Medicine at the University of Bonn where he heads the HIV outpatient clinic. Sanjay Bhagani is a Consultant Physician in Infectious Diseases and HIV Medicine at the Royal Free Hospital, London where he leads a multi-disciplinary HIV/hepatitis co-infection clinic and is an honorary Senior Lecturer at UCL.

## Pre-publication history

The pre-publication history for this paper can be accessed here:

http://www.biomedcentral.com/1741-7015/11/234/prepub

## Note

This article is part of the cross journal collection HIV 30 years on. Other articles in this series can be found at http://www.biomedcentral.com/series/HIV_30.
